# Differences in neural activation as a function of risk-taking task parameters

**DOI:** 10.3389/fnins.2013.00173

**Published:** 2013-09-30

**Authors:** Eliza Congdon, Angelica A. Bato, Tom Schonberg, Jeanette A. Mumford, Katherine H. Karlsgodt, Fred W. Sabb, Edythe D. London, Tyrone D. Cannon, Robert M. Bilder, Russell A. Poldrack

**Affiliations:** ^1^Department of Psychiatry, Semel Institute for Neuroscience and Human Behavior, University of California Los AngelesLos Angeles, CA, USA; ^2^Center for Neurobehavioral Genetics, Semel Institute for Neuroscience and Human Behavior, University of California Los AngelesLos Angeles, CA, USA; ^3^Department of Psychiatry, Zucker Hillside Hospital, North Shore-LIJQueens, NY, USA; ^4^Imaging Research Center, University of Texas at AustinAustin, TX, USA; ^5^Department of Psychology, University of Texas at AustinAustin, TX, USA; ^6^Department of Molecular and Medical Pharmacology, University of California Los AngelesLos Angeles, CA, USA; ^7^Department of Psychology, Yale UniversityNew Haven, CT, USA; ^8^Department of Psychology, University of California Los AngelesLos Angeles, CA, USA; ^9^Department of Neurobiology, University of Texas at AustinAustin, TX, USA

**Keywords:** functional impulsivity, dysfunctional impulsivity, risky decision-making, naturalistic risk-taking, ART, BART

## Abstract

Despite evidence supporting a relationship between impulsivity and naturalistic risk-taking, the relationship of impulsivity with laboratory-based measures of risky decision-making remains unclear. One factor contributing to this gap in our understanding is the degree to which different risky decision-making tasks vary in their details. We conducted an fMRI investigation of the Angling Risk Task (ART), which is an improved behavioral measure of risky decision-making. In order to examine whether the observed pattern of neural activation was specific to the ART or generalizable, we also examined correlates of the Balloon Analog Risk Taking (BART) task in the same sample of 23 healthy adults. Exploratory analyses were conducted to examine the relationship between neural activation, performance, impulsivity and self-reported risk-taking. While activation in a valuation network was associated with reward tracking during the ART but not the BART, increased fronto-cingulate activation was seen during risky choice trials in the BART as compared to the ART. Thus, neural activation during risky decision-making trials differed between the two tasks, and this observation was likely driven by differences in task parameters, namely the absence vs. presence of ambiguity and/or stationary vs. increasing probability of loss on the ART and BART, respectively. Exploratory association analyses suggest that sensitivity of neural response to the magnitude of potential reward during the ART was associated with a suboptimal performance strategy, higher scores on a scale of dysfunctional impulsivity (DI) and a greater likelihood of engaging in risky behaviors, while this pattern was not seen for the BART. Our results suggest that the ART is decomposable and associated with distinct patterns of neural activation; this represents a preliminary step toward characterizing a behavioral measure of risky decision-making that may support a better understanding of naturalistic risk-taking.

## Introduction

Risky decision-making has important implications for understanding a number of potentially harmful behaviors, ranging from those linked to clinical disorders that place a significant burden on both the affected individual and society (e.g., substance dependence) to behaviors that are not linked to specific diagnoses but carry potentially adverse outcomes (e.g., fast driving). While trait impulsivity plays a role in risky decision-making and predicts naturalistic risk-taking (that is, an individual's likelihood of engaging in real-world activities that involve potential negative outcomes), there is not yet a clear pattern of individual differences in risky decision-making (as measured behaviorally) and impulsivity that can predict naturalistic risk-taking. This lack of understanding is likely driven by the complex nature of decision-making itself, and the wide variability in behavioral tasks designed to assess risky decision-making.

In laboratory-based decision-making tasks, participants are required to choose between two options of varying risk (defined as variance over possible outcomes with known probabilities) and/or ambiguity (defined by the uncertainty of outcome probabilities). As reviewed in Schonberg et al. (2011), there is a further distinction between economic tasks (which have traditionally been used in neuroeconomics research) and naturalistic risk-taking tasks. While economic tasks are easily decomposable, performance on such tasks is not reliably correlated with naturalistic risk-taking; in contrast, tasks on which the associated performance is correlated with self-reported naturalistic risk-taking do not generally lend themselves to clean cognitive decomposition.

For example, one laboratory test of naturalistic decision-making is the Balloon Analog Risk Taking (BART) task (Lejuez et al., 2002), in which participants inflate a series of simulated balloons and chose between continuing to play (with the increased probability of a loss) or cashing out (to keep their potential accumulated earnings). Performance on the BART has been associated with self-reported naturalistic risk-taking, as exemplified by substance abuse and alcohol-related problems (Lejuez et al., 2002; Aklin et al., 2005; Skeel et al., 2008; Fernie et al., 2010; Weafer et al., 2011), although this has not been universally true (Dean et al., 2011; Courtney et al., 2012). A limitation of this task is that loss probability is confounded with changing expected value (as both potential gains and losses, and loss probability, increase across trials).

In the Angling Risk Task (ART) (Pleskac, 2008), which was developed to address some of the problems with interpretation of risk-taking on the BART, participants play in a simulated fishing tournament and, similar to the instructions on the BART, they choose between continuing to play vs. cashing out. In one version of the ART, the probability of loss is known and constant across trials, as participants can see the unchanging distribution of good-to-bad fish in the pond on every trial. This design makes the task more decomposable than the BART, as the only objectively changing variables are total potential gains and losses. In a previous study, risk-taking on the ART was associated with self-reported drug use (Pleskac, 2008), suggesting that this task was able to maintain the affective features that appear to be essential for predictive validity of naturalistic risk-taking (Schonberg et al., 2011).

Here, we set out to investigate the neural correlates of risk-taking on the ART in a sample of healthy adults and compare neural activation between ART and BART in order to test the influence of task parameters on relationships with individual difference measures. Given the lack of ambiguity as well as the stationary probability of loss on the ART, we predicted that this task would be decomposable as evidenced by distinct patterns of activation (e.g., increased activation in reward-related regions on increasingly risky trials), while this pattern would be relatively obscured by the presence of ambiguity (and increasing probability of loss) on the BART. In follow-up exploratory analyses, we also examined whether the ART—both in terms of behavior and neural activation—is also sensitive to individual differences in trait impulsivity and naturalistic risk-taking. Although our findings of a pattern of individual differences based on the ART data are limited by the small sample, our findings of significant differences between activation elicited during the ART as compared to BART provide support for the influence of these specific task parameters on resulting patterns of neural activation.

## Materials and methods

### Participants

All participants were recruited from the Los Angeles area as part of the Consortium for Neuropsychiatric Phenomics at UCLA (www.phenomics.ucla.edu), in which they completed extensive neuropsychological testing. A portion of the sample also took part in two separate fMRI sessions; data collected during these scan sessions (which included the BART) are the focus of analyses presented here. All candidates had telephone screening followed by additional in-person screening. After receiving a thorough explanation, all participants gave written informed consent according to the procedures approved by the University of California Los Angeles Institutional Review Board.

Participants were men or women ages 21–50 years; of the NIH racial/ethnic category either White, not Hispanic or Latino, or Hispanic or Latino, of any racial group. Their primary language was either English or Spanish, and they had completed at least 8 years of formal education. They were required to have no significant medical illness; to be adequately cooperative to complete assessments; and to have visual acuity of 20/60 or better. Participants were excluded if they had lifetime diagnoses of Schizophrenia or Other Psychotic Disorder, Bipolar I or II Disorder; or current Major Depressive Disorder, suicidality, Anxiety Disorder (Obsessive Compulsive Disorder, Panic Disorder, Generalized Anxiety Disorder, Post-Traumatic Stress Disorder), Substance Abuse/Dependence, or ADHD. Diagnoses followed the Diagnostic and Statistical Manual of Mental Disorders, Fourth Edition—Text Revision (American Psychiatric Association, 2000), and were based on the Structured Clinical Interview for DSM-IV [SCID-I; (First et al., 2004)], supplemented by the Adult ADHD Interview. Additional exclusion criteria for participants in the imaging portion of the study included left-handedness, pregnancy, history of head injury with loss of consciousness or cognitive sequelae, or other contraindications to scanning (e.g., claustrophobia, metal in body, body too large to fit in scanner).

A subsample of the participants that were scanned took part in a third fMRI session, which included 1 h of behavioral testing and 1 h of scanning on the same day including the ART task. Eligible English-speaking participants between the ages of 21 and 40 were recruited from the parent study if they successfully completed all previous testing sessions. A total of 28 participants were recruited and completed the third scan session. Of these, 23 were included in the final analyses [mean age 25.65 years (*SD* = 4.43), range 21–39; 13 women], after 5 individuals were excluded for failing to perform either of the tasks as instructed (*N* = 2), excessive head motion (exceeding 3 mm in any direction) during scanning (*N* = 2), or poor quality of the high-resolution anatomical image collected (*N* = 1).

### fMRI tasks

**Figure 1:**
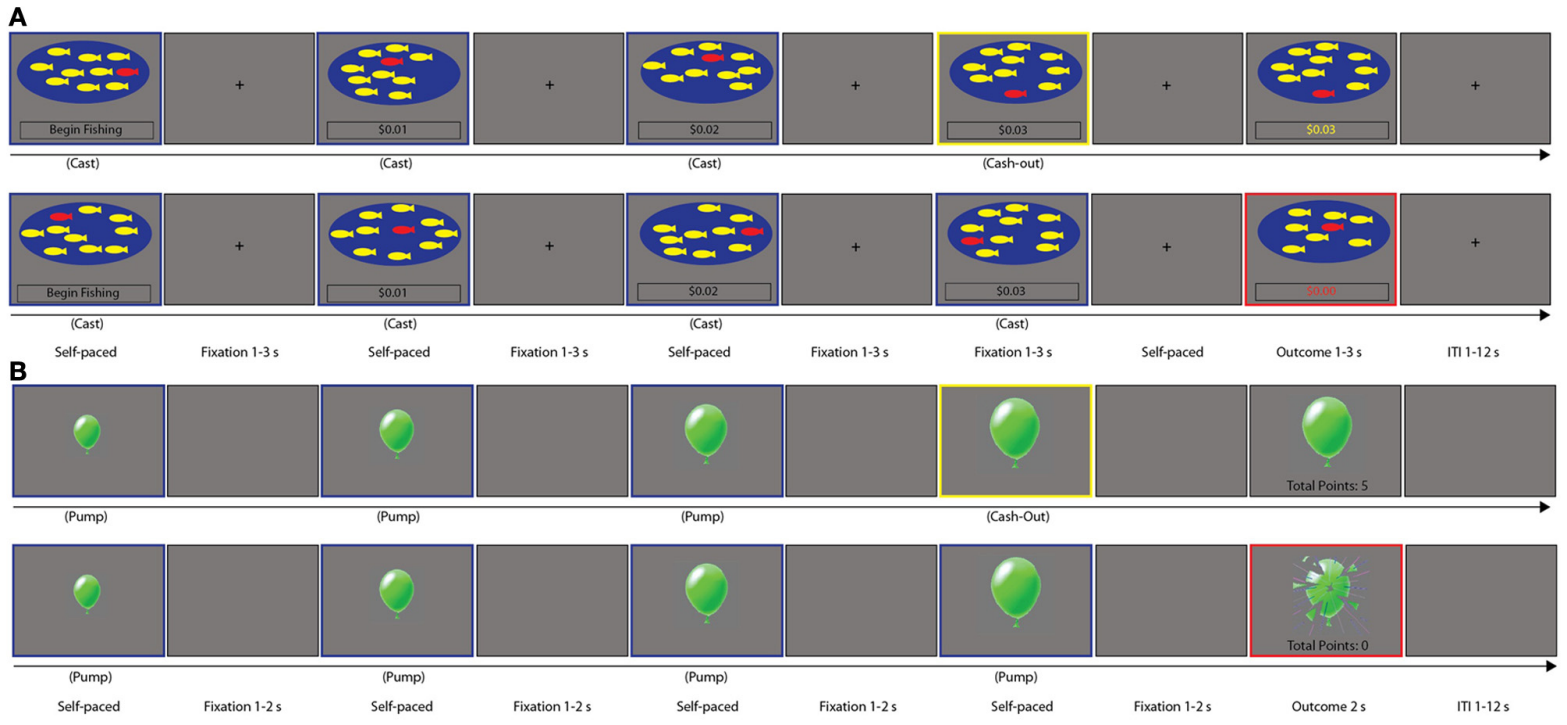
**Task Schematic of the (A) Angling Risk Task (ART) and (B) Balloon Analog Risk Taking task (BART).** For both tasks, Risky choice trials are enclosed in blue, Cash-out trials are enclosed in yellow, and Loss trials are enclosed in Red; trials are indicated as such for the purpose of illustration only, and were not colored as such during task presentation.

Participants performed an fMRI-adapted version of the ART during the testing session that is the focus of our primary analysis. In addition, participants had completed the BART, as part of the parent study, during a previous scan session. Both the ART and BART tasks are sequential measures of dynamic risk-taking behavior and include similar trial types: Risky Choices, Cash-outs and Losses (with Loss trials defined as loss of accumulated potential gain). Across trials, participants chose between a sure outcome (by selecting the Cash-out option and ending the round) and a risky outcome (by making a Risky Choice and continuing to play in the round); as such, the outcome of each round was *not* pre-determined. A schematic of each task is presented in Figure 1. Each task included a series of rounds, each including potentially multiple Risky Choice trials and ending with either a Cash-out trial or a Loss trial (that is, each task included a number of rounds, with each round including multiple trials). Risky Choice trials (trials in a blue frame in Figure 1) are defined as those in which the participant chooses to continue playing a round, by either casting the fishing rod again (ART) or pumping the balloon again (BART). Cash-out trials (trials in a yellow frame in Figure 1) are defined as those in which the participant chooses to save the accumulated earnings and end the given round. Loss trials (trials in a red frame in Figure 1) are defined as those in which the participant experiences a loss of potential accumulated earnings (by either catching the bad fish in the ART or the balloon exploding in the BART) and mark the end of the given round.

#### ART

In the ART (Pleskac, 2008), which in its design was based on the BART, participants were allowed to fish, in the context of a fishing tournament, out of a pond with nine yellow fish and one red fish (Figure 1A). On each trial, participants chose to fish or cash out to collect their accumulated earnings for that round. After a trial in which the participant chose to cast and then caught a yellow fish, the participant earned $0.01 (which was displayed on the screen) and was able to cast again as part of that round; however, if the participant caught a red fish, all earnings for that round were forfeited, a $0.00 total was displayed, and the next round began. Alternatively, if the participant chose to cash out, his or her accumulated earnings for that round were displayed before the task moved onto the next round. As illustrated in the top panel of Figure 1A, the potential accumulated earnings on the fourth trial of this example round was $0.03, after the participant completed three successful Risky Choice cast trials. The top panel of Figure 1A represents a Cash-out decision on the fourth trial, where the participant would then receive the accumulated earnings of $0.03 in the subsequent outcome trial. The bottom panel of Figure 1A represents a Loss event on the fifth trial, after the participant completed four Risky Choice trials; the round ends in a Loss event, with the participant losing all potential accumulated earnings for that round. In the “sunny day, catch-and-release” version of the ART used here [rather than other versions of the task tested by Pleskac (2008)], participants chose to fish out of a clear pond, meaning that they could see the distribution of yellow-to-red fish and the distribution of fish remained the same on every trial. The probability of the participant catching a red fish on each trial was based on the distribution of red-to-yellow fish in the pond (one out of ten), meaning that on each trial, the probability of a loss was explicit and stationary. There was no limit to the number of trials that could occur within a round. The task was self-paced: the outcome trial was displayed for a variable duration (1–3 s, average 2 s). Each trial was separated by presentation of a fixation point for a variable duration (1–3 s, average 2 s); each round was separated by presentation of a fixation for variable duration (1–12 s, average 4 s).

#### BART

In the BART (Lejuez et al., 2002), participants were allowed to pump a series of green (experimental) and white (control) balloons (Figure 1B). On each trial, participants chose to pump the balloon or cash out and collect their accumulated earnings for that round. For experimental balloons, after a trial in which the participant pumped the balloon without an explosion occurring, an image of a larger balloon was presented, the participant earned 5 points, and was able to pump again or cash-out. After a trial in which the participant chose to cash out, the accumulated earnings for that round were displayed and the task progressed to the next round. On an explosion trial (necessarily following a Risky choice trial), an exploded balloon was pictured, the participant received no points for that round, and the task progressed to the next round. As illustrated in the top panel of Figure 1B, the potential accumulated points on the fourth trial of this example round was 15, after the participant completed three successful Risky Choice pump trials. The top panel of Figure 1B represents a Cash-out decision on the fourth trial, where the participant would then receive the accumulated 15 points in the subsequent outcome trial. The bottom panel of Figure 1B represents a Loss event on the fifth trial, after the participant completed four Risky Choice trials; the round ends in a Loss event, with the participant losing all potential accumulated earnings for that round. In this version of the BART, the number of pumps to explosion was randomly drawn from a uniform distribution of numbers from 1 to 12, which was the maximum number of pumps possible before an explosion or successful end of a round. Under these conditions, participants experienced the probability as non-stationary, as the likelihood of a loss event increased with each trial in a round, and no information was provided to subjects about the probability of explosion. Participants also responded to control balloons, which increased in size on successive trials, but were not associated with earning of points and did not explode. Only data from experimental balloons are presented below. An outcome trial (following a Cash-out choice or a Loss event) was displayed for a fixed duration of 2 s. Each trial was separated by presentation of a blank screen for a variable duration (1–2 s, average 1.5 s); each round was separated by presentation of a blank screen for variable duration (1–12 s, average 4 s).

All participants received brief training on each task immediately before scanning. Each participant viewed the task stimuli through MRI-compatible goggles, responded with his or her right hand on an MR-compatible button box in the scanner, and performed one run of each task while in the scanner. The presentation and timing of all stimuli and response events were achieved using Matlab (Mathworks) and the Psychtoolbox (www.psychtoolbox.org) on an Apple Powerbook. Participants received earnings based on performance in the ART at the end of their testing session, but did not receive payment for performance on the BART.

### Image acquisition and analysis

Data were collected using a 3T Siemens Trio MRI scanner. For each task run, functional T2^*^-weighted echoplanar images (EPIs) were collected with the following parameters: slice thickness = 4 mm, 34 slices, *TR* = 2 s, *TE* = 30 ms, flip angle = 90°, matrix 64 × 64, FOV = 192 mm. For the ART, 270 EPIs were collected; for the BART, 267 EPIs were collected. Additionally, a T2-weighted matched-bandwidth high-resolution anatomical scan (same slice prescription as EPI) and MPRAGE were collected. The parameters for MPRAGE were the following: *TR* = 1.9 s, *TE* = 2.26 ms, FOV = 250, matrix = 256 × 256, sagittal plane, slice thickness = 1 mm, 176 slices.

### fMRI data analysis

Analyses were performed using tools from the FMRIB software library (www.fmrib.ox.ac.uk/fsl), version 4.1. The first 2 volumes from each scan were discarded to allow for T1 equilibrium effects. For each scan, images for each participant were realigned to compensate for small head movements (Jenkinson and Smith, 2001). Data were spatially smoothed using a 5-mm, full-width-half-maximum Gaussian kernel. The data were filtered in the temporal domain using a non-linear high-pass filter with a 66 s cutoff. A three-step registration process was used in which EPI images were first registered to the matched-bandwidth high-resolution scan, then to the MPRAGE structural image, and finally into standard [Montreal Neurological Institute (MNI)] space, using non-linear transformations (Andersson et al., 2007a,b).

Standard model fitting was conducted for all subjects. We modeled the mean activation associated with Risky choice, Loss and Cash-out and also evaluated how these activations varied according to reaction time (Risky choice and Cash-out), as well as the associated reward-value of the current trials (all three trial types, although Loss trials are not considered here, as Loss was experienced similarly between tasks and there were few Loss trials in both tasks); for further description of this model set-up, see Schonberg et al. (2012). The mean activation for each task was modeled as a 1 s trial at the time of each event for all three event types. Reaction time (RT) for Risky choice and Cash-out was modeled using the same onset times as the mean activation, but the duration was the participant's RT for the trial of interest. To preserve the meaning of the mean activation regressor, the RT regressors were orthogonalized with respect to the mean regressors within event type. Trial values (i.e., potential accumulated money for ART, potential accumulated balloon points for BART) for all three events were modeled through a parametrically modulated regressor (although the parametric Risky choice contrast is only considered here), again with the same onset, but with a modulation value determined by the current trial's potential accumulated money/points after mean centering over potential accumulated money/points within the round being evaluated. Looking at Figure 1A, the parametric regressor for the three Risky Choice trials in the ART would be weighted by the value presented below each pond, after mean centering for the average amount reached in that round; looking at Figure 1B, the parametric regressor for the three Risky Choice trials in the BART would be weighted by the value associated with each Risky Choice trial, after mean centering over the average amount reached in that round. As both tasks included a set of trials within a series of rounds, with each Risky Choice trial associated with an increasing potential accumulated value within a given round, we were therefore able to examine parametrically modulated activation for Risky Choice trials. This means that for both ART and BART, we examined the mean Risky choice, mean Cash-out, and mean Loss contrasts, as well as the Risky choice Parametric contrast. In addition, Control trials were modeled separately for the BART.

In all cases, events were convolved with a double gamma hemodynamic function to create the regressor. Null events were not modeled and, therefore, constituted an implicit baseline. The six motion parameters and temporal derivatives of all regressors were included as covariates of no interest to improve statistical sensitivity. For each participant, four contrasts were computed: Risky choice vs. Null, Risky choice Parametric vs. Null, Cash-out vs. Null, and Loss vs. Null. As we were primarily interested in the average activation for each trial separately, and in the relationship between activation during each trial type and our individual differences measures, we examined contrasts of each trial type relative to an implicit baseline (i.e., Null). Furthermore, a direct comparison between trial types given the design of both tasks is difficult as a round includes multiple Risky Choice trials but either one Cash-out or one Loss trial, resulting in an unbalanced number of trials across trial types (see Table 1 for the number of trials).

**Table 1:** **Descriptive statistics of sample demographics and study measures**.

**Variable**	**Mean**	***SD***	**Minimum**	**Maximum**
Age (years)	25.65	4.43	21.00	39.00
DI	1.48	2.56	0.00	9.00
FI	5.91	2.47	1.00	10.00
DOSPERT total score	98.22	23.21	57.00	146.00
ART: total rounds	25.78	4.00	18.00	30.00
ART: Risky choice trials	112.48	29.51	57.00	158.00
ART: Cash-out trials	11.78	3.98	5.00	19.00
ART: Loss trials	13.96	2.72	8.00	18.00
ART: Mean adjusted presses	7.22	3.94	2.00	16.83
ART: Total amount earned (dollars)	0.71	0.20	0.38	1.18
ART: Coefficient of variation	0.38	0.19	0.08	1.07
ART: Total number presses	149.91	23.72	106	188
BART: Total rounds	27.61	2.92	22.00	35.00
BART: Risky choice trials	70.43	10.57	55.00	99.00
BART: Cash-out trials	10.09	3.41	2.00	17.00
BART: Loss trials	8.30	3.02	4.00	14.00
BART: Mean adjusted presses	4.58	1.24	2.23	8.40
BART: Total amount earned (points)	211.30	52.92	70.00	320.00
BART: Coefficient of variation	0.27	0.11	0.14	0.60
BART: Total number presses	143	9.83	122	161

*DI, Dysfunctional Impulsivity; FI, Functional Impulsivity; DOSPERT, Domain-Specific Risk-Taking Scale; ART, Angling Risk Task; BART, Balloon Analog Risk Task; SD, standard deviation*.

Output from the subject-specific analyses was then analyzed using a mixed-effects model with FLAME. Higher-level analyses included the four group-level contrasts defined at the subject level, for ART and BART separately. Primary analyses are focused on the group-level contrasts of the ART. Conjunction maps were created for Risky choice vs. Null and Risky choice Parametric vs. Null contrasts in order to identify the regions of overlapping activation in both tasks. Finally, paired *t*-tests were conducted to contrast ART vs. BART directly, for each of these contrasts. All group-level statistics images were thresholded with a cluster-forming threshold of *z* > 2.3 and a cluster probability of *p* < 0.05, corrected for whole-brain multiple comparisons using Gaussian random field theory. The search region included 273,797 voxels for ART and 272,763 voxels for BART. Brain regions were identified using the Harvard-Oxford cortical and subcortical probabilistic atlases, and all locations of activations are reported using MNI coordinates. For visualization of results, statistical maps were projected onto an average cortical surface with the use of multifiducial mapping using CARET software (Van Essen, 2005). For reporting of clusters, we used the cluster command in FSL. Anatomical localization within each cluster was obtained by searching within maximum likelihood regions from the FSL Harvard-Oxford probabilistic atlas to obtain the maximum z-statistic and MNI coordinates within each anatomical region contained within a cluster.

In addition, in order to examine the relationship between individual differences and activation as measured using fMRI, we defined two anatomically-based regions of interest [the right nucleus accumbens (NAcc) and ventromedial prefrontal cortex (vmPFC)] following inspection of group contrasts, in addition to an extensive literature supporting the role of these regions in reward processing (Liu et al., 2011); note that these ROIs were defined prior to any individual difference analyses on this dataset, and thus are fully independent. ROIs were defined using the FSL Harvard-Oxford probabilistic atlas (thresholded at 25%): we used the existing NAcc mask and created a mask to encompass the medial PFC by combining Harvard-Oxford regions (frontal pole, frontal medial cortex, paracingulate gyrus, and subcallosal cortex) falling between *x* = 14 and *x* = −14 and *z* < 0. ROIs were then used to extract average percent signal change values corresponding to a 1-s stimulus convolved with a double-gamma HRF from the Risky choice Parametric contrast (following Mumford and Poldrack, 2007), representing the average across all voxels included in the anatomically-defined ROI. In this way, we restricted our analyses to activation in the ART and BART Risky choice Parametric contrasts that fell within these anatomically-defined regions. Percent signal change values were then correlated with our individual differences measures using R statistical software.

### Individual difference measures for exploratory analyses

#### Self-report questionnaires

Participants provided demographic data and completed the Dickman Scale of Functional and Dysfunctional Impulsivity (DI), which consists of 23 items assessing functional and DI factors, with reported Cronbach's alphas of 0.83 and 0.86, respectively (Dickman, 1990). Functional Impulsivity (FI) is conceptualized as a tendency to act with relatively little forethought when such a strategy is advantageous, and is evaluated with questions such as “I am good at taking advantage of unexpected opportunities, where you have to do something immediately or lose your chance” and “I would enjoy working at a job that required me to make a lot of split-second decisions.” DI is conceptualized as a tendency to act with less forethought than most people of equal ability when this tendency is a source of difficulty, and is assessed with questions such as “I frequently buy things without thinking about whether or not I can really afford them” and “I often say and do things without considering the consequences.” Total scores were calculated separately for FI and DI.

Participants also completed the Domain-Specific Risk-Taking Scale (DOSPERT), which assesses individual differences in work-related or personal decisions that involve risk and uncertainty (Weber et al., 2002; Blais and Weber, 2006). The scale assesses the likelihood of engaging in thirty risky behaviors across five domains (ethics, financial, health/safety, recreational, and social domains), and uses a 7-point rating scale ranging from 1 (Extremely Unlikely) to 7 (Extremely Likely). Sample items include “Having an affair with a married man/woman” (Ethical); “Betting a day's income at the horse races” (Financial); “Engaging in unprotected sex” (Health/Safety); “Taking a weekend sky-diving class” (Recreational); and “Disagreeing with an authority figure on a major issue” (Social). The DOSPERT questionnaire was administered to provide a single index of risk-taking propensity, which was calculated as the total score based on reported likelihood of engaging in the 30 behaviors across five domains. We used the total score of risk-taking propensity, rather than domain subscale scores, given our sample size, as well as the lack of evidence suggesting differences in ART or BART performance as a function of risk-taking across domains (e.g., Recreational vs. Social).

#### Behavioral data analysis

Three primary measures were calculated on the basis of task performance for both the ART and the BART: Adjusted Presses, Total Amount Earned, and Coefficient of Variation. The measure of “*Adjusted Presses”* reflected the mean number of choice responses (Cast for ART and Pump for BART) made on rounds that do not include a loss event (which otherwise would artificially restrict the range of risk behavior on that round). Adjusted Presses is considered an index of risk-taking (Lejuez et al., 2002), although it is alternatively considered to be an index of performance strategy (Jentsch et al., 2010; Ashenhurst et al., 2011), as it reflects how many trials—on average—that the participant is willing to accept before cashing-out. “*Total Amount Earned”* is the total amount of money and points earned in the ART and BART, respectively, which reflects the subject's ability to maximize reward receipt. The “*Coefficient of Variation,”* which is also calculated from rounds that don't include a loss event, is the standard deviation of the participant's Adjusted Presses divided by the mean of Adjusted Presses and reflects the subject's variability in responding. It has been suggested that a high coefficient of variation in a rodent model of the BART reflects combinations of both higher than optimal and lower than optimal trial completions and has been shown to index response strategy (Jentsch et al., 2010). Pearson's correlations were computed using R statistical software (R 2.10.1) (http://www.r-project.org) in order to test the association between behavioral performance, Dickman scale factors, and DOSPERT total scores. In order to address potential effects of outliers, follow-up robust regression analyses were computed when necessary. We present our correlation results uncorrected for multiple comparisons given the exploratory nature of these analyses and our small sample.

## Results

Given that our goal was to characterize the ART, and then to compare neural activation elicited between the ART and BART, we present results from our analyses of the ART imaging data, followed by a comparison of the ART and BART contrasts. Note that we present only the results of the BART analyses in relation to our hypotheses about the ART, given recent and more extensive analyses of the BART alone in other datasets (Schonberg et al., 2012; Galvan et al., 2013).

### ART group contrasts

**Figure 2:**
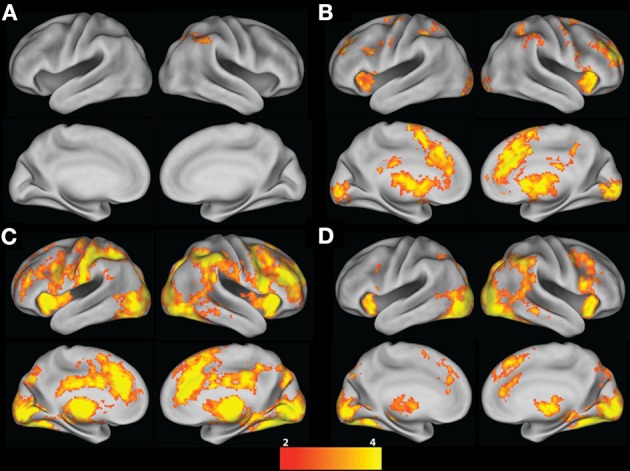
**ART group contrasts. (A)** Risky choice; **(B)** Risky choice Parametric; **(C)** Cash-out; and **(D)** Loss contrasts (vs. Baseline). All contrasts are corrected for whole-brain multiple comparisons; statistical maps were projected onto an average cortical surface using CARET (Right hemisphere = Right side of the image). The color scale represents z-score.

Inspection of activation resulting from the mean Risky choice contrast of the ART revealed activation only in right posterior parietal and occipital regions (Table 2 and Figure 2A). In contrast, the Risky choice Parametric contrast (which indexed increasing activation across pumps within a round) revealed activation in a paracingulate cluster spreading through the pre-supplementary motor area (preSMA), superior frontal gyrus (SFG), and anterior cingulate cortex (ACC), and ventrally to the subcallosal and vmPFC, also extending bilaterally through the inferior frontal regions, precentral gyri, and basal ganglia (including NAcc, striatum, and thalamus) (Table 2 and Figure 2B). Activation was also seen in bilateral frontal regions, the posterior cingulate, bilateral posterior parietal regions, and occipital cortex. There was no significant activation that was negatively associated with pumps within a round.

**Table 2:** **ART group contrasts**.

**Brain region**	**Hemisphere**	**Voxels**	**Max z-statistic**	***x***	***y***	***z***
**ART RISKY CHOICE**						
Superior parietal cortex, lateral occipital cortex	R	623	3.85	28	−54	46
**ART RISKY CHOICE PARAMETRIC**						
Paracingulate, preSMA, SFG, ACC, subcallosal cortex, PCC, precuneus, medial and lateral frontal pole, thalamus, caudate, putamen, NAcc, globus pallidus, insula, frontal orbital cortex, vmPFC, MFG, precentral gyrus, cerebellum, occipital cortex	R/L	37183	5.83	2	22	38
Posterior parietal cortex	R	748	3.93	50	−40	52
Posterior parietal cortex	L	520	4.07	−40	−42	48
**ART CASH-OUT**						
Posterior parietal cortex, occipital cortex, precuneus, cerebellum, PCC, preSMA, SFG, paracingulate, ACC, frontal pole, thalamus, caudate, putamen, NAcc, globus pallidus, insula, frontal operculum, IFG, frontal orbital cortex, MFG, precentral gyrus, frontal pole, middle and inferior temporal gyrus	R/L	79621	5.63	50	−34	44
**ART LOSS**						
Occipital cortex, cerebellum, precuneus, posterior parietal cortex, middle temporal gyrus, parahippocampal gyrus, thalamus, caudate	R/L	26384	6.14	28	−70	−14
Frontal orbital cortex, insula, IFG, MFG, precentral gyrus	R	2579	4.84	32	18	−14
SFG, preSMA, paracingulate, ACC	R/L	2458	4.18	4	32	46
Insula, frontal orbital cortex, frontal operculum, IFG, precentral gyrus	L	969	4.36	−34	16	−8

*Voxels: number of activated voxels per cluster (or region within cluster); z-stat: maximum z-statistic for each cluster; x, y, z are MNI coordinates for the peak of each cluster. ART, Angling Risk Task; R, right; L, left; preSMA, presupplementary motor area; SFG, superior frontal gyrus; ACC, anterior cingulate cortex; PCC, posterior cingulate cortex; NAcc, nucleus accumbens; vmPFC, ventromedial prefrontal cortex; MFG, middle frontal gyrus; IFG, inferior frontal gyrus*.

Inspection of the mean Cash-out contrast of the ART, when participants chose to end the round and collect their accumulated earnings, revealed widespread activation spreading through the cingulate/SMA/SFG, bilateral inferior frontal gyri spreading through frontal poles and structures of the basal ganglia (including thalamus, caudate, putamen, NAcc, and globus pallidus), in addition to activation in the precentral gyrus, middle frontal gyrus (MFG), frontal pole, and middle and inferior temporal gyrus (Table 2 and Figure 2C). Inspection of activation from the mean Loss contrast of the ART, when participants were presented with a red fish and lost their accumulated earnings for that round, also revealed activation in the ACC spreading through the paracingulate, preSMA and SFG, activation in bilateral thalamus, caudate, parahippocampal gyrus, IFG/insula/frontal orbital regions, in addition to posterior parietal and occipital regions (Table 2 and Figure 2D).

### ART vs. BART group contrasts

The results of our primary analyses suggest that the version of the ART used here engages brain regions known to be involved in reward processing (for the Risky choice Parametric contrast). In order to examine whether this pattern was specific to the ART (and its task parameters), or generalizable to the BART (which has a less decomposable structure), we examined neural and behavioral correlates of the task in the same sample of participants.

**Figure 3:**
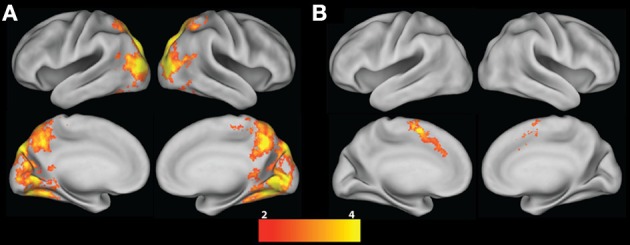
**Risky choice contrast. (A)** ART vs. BART group contrast; **(B)** BART vs. ART group contrast. All contrasts are corrected for whole-brain multiple comparisons; statistical maps were projected onto an average cortical surface using CARET (Right hemisphere = Right side of the image). The color scale represents z-score.

Given the lack vs. presence of ambiguity and changes in loss probability in the ART and BART, respectively, we first compared group-level mean Risky choice contrasts between tasks to test the hypothesis that the addition of ambiguity and changes in probability of gain and loss in the BART Risky choice trials would be evident in increased neural activation in frontal, cingulate, and striatal regions. We examined the conjunction of activation for both tasks, which revealed that there was no overlapping activation for the mean Risky choice contrast between BART and ART. We directly contrasted group maps between tasks, revealing significantly greater activation in bilateral occipital regions in the ART vs. BART mean Risky choice contrast, which extended into superior parietal regions (Table 3 and Figure 3A). We found significantly greater activation in a fronto-cingulate cluster in the BART vs. ART mean Risky choice contrast, with activation extending from the SMA, preSMA, SFG, and paracingulate through the ACC (Table 3 and Figure 3B).

**Table 3:** **Contrasts between ART and BART tasks**.

**Brain region**	**Hemisphere**	**Voxels**	**Max z- statistic**	***x***	***y***	***z***
**ART vs. BART RISKY CHOICE**						
Occipital cortex, precuneus, superior parietal cortex	R/L	21169	6.14	30	−82	20
**BART vs. ART RISKY CHOICE**						
preSMA, SMA, paracingulate, ACC, SFG	R/L	936	4.68	−4	0	54
**ART vs. BART RISKY CHOICE PARAMETRIC**						
Caudate, putamen, NAcc, thalamus, preSMA, paracingulate, ACC, frontal orbital cortex, frontal pole, vmPFC, insula, IFG, frontal operculum	R/L	7505	4.67	14	8	4
Occipital pole, cerebellum	R/L	4658	4.59	20	−92	−8
Frontal pole, MFG	R	1505	4.02	32	58	24
Cerebellum	R/L	620	3.45	−4	−54	−38
MFG, SFG, precentral gyrus	R	338	3.60	34	10	60
**BART vs. ART RISKY CHOICE PARAMETRIC**						
(no greater activation)	–	–	–	–	–	–

*Voxels: number of activated voxels per cluster (or region within cluster); z-stat: maximum z-statistic for each cluster; x, y, z are MNI coordinates for the peak of each cluster. BART, Balloon Analog Risk Task; ART, Angling Risk Task; R, right; L, left; preSMA, presupplementary motor area; SMA, supplementary motor area; ACC, anterior cingulate cortex; SFG, superior frontal gyrus; NAcc, nucleus accumbens; vmPFC, ventromedial prefrontal cortex; IFG, inferior frontal gyrus; MFG, middle frontal gyrus; SFG, superior frontal gyrus*.

**Figure 4:**
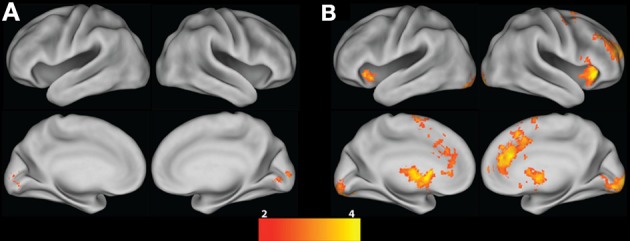
**Risky choice Parametric contrast. (A)** Conjunction between BART and ART; **(B)** ART vs. BART group contrast. All contrasts are corrected for whole-brain multiple comparisons; statistical maps were projected onto an average cortical surface using CARET (Right hemisphere = Right side of the image). The color scale represents z-score.

We next compared group-level Risky choice Parametric contrasts between tasks to test the hypothesis that the lack of ambiguity (and stationary gain and loss probabilities) in the ART Risky choice Parametric contrast—as compared to the BART Risky choice parametric contrast—would be evident in increased activation within a valuation network (including the ventral striatum and medial prefrontal cortex). A conjunction of activation for both tasks revealed overlapping activations in only bilateral occipital regions between BART and ART Risky choice Parametric contrasts (Table 3 and Figure 4A). The ART, as compared to BART, Risky choice Parametric contrast revealed significantly greater activation in the bilateral NAcc, striatum, globus pallidus, thalamic nuclei, insula, IFG, and frontal orbital cortex, in addition to a paracingulate cluster extending to the vmPFC, frontal pole, and ACC (Table 3 and Figure 4B). In contrast, no region showed significantly greater activation for the Risky choice Parametric contrast for BART as compared to ART.

### Exploratory individual differences analyses

We conducted a number of exploratory follow-up analyses in order to further characterize the pattern of individual differences evident during performance of the ART, as compared to the BART. These analyses are strictly exploratory and caution must be used when interpreting them given our small sample size. Descriptive statistics of sample demographics, self-report scores and task performance are presented in Table 1, and cross-correlation matrices are presented in Table 4.

**Table 4:** **Correlations among individual differences measures**.

	**DI**	**DOSPERT**	**Adjusted presses**	**Total amount earned**	**Coefficient of variation**	**ART NAcc %sc**	**ART vmPFC %sc**
**ART**							
FI	0.22	0.04	0.18	−0.02	−0.10	−0.27	−0.19
DI	–	0.27	−0.29	−0.19	−0.11	0.54^*^	0.16
DOSPERT	–	–	−0.41^†^	−0.53^*^	−0.17	0.35	0.39^†^
Adjusted presses	–	–	–	0.74^*^	0.15	−0.59^*^	−0.38^†^
Total amount earned	–	–	–	–	0.14	−0.47^*^	−0.39^†^
Coefficient of variation	–	–	–	–	–	0.06	0.17
ART NAcc %sc	–	–	–	–	–	–	0.52^*^
			**Adjusted presses**	**Total amount earned**	**Coefficient of variation**	**BART NAcc %sc**	**BART vmPFC %sc**
**BART**							
FI			0.39+	0.04	−0.52^*^	0.05	0.007
DI			0.03	0.23	0.17	0.20	−0.23
DOSPERT			−0.21	0.03	0.08	−0.49^*^	−0.50^*^
Adjusted presses			–	0.02	−0.42^*^	0.32	0.22
Total amount earned			–	–	−0.19	0.20	−0.15
Coefficient of variation			–	–	–	−0.19	−0.04
BART NAcc %sc			–	–	–	–	0.56

* p < 0.05;

† *Trend-level association (p-value between 0.05 and 0.07). %sc = percent signal change*.

#### Individual differences analysis of behavioral data

In terms of ART performance measures, mean Adjusted Presses and Total Amount Earned were positively correlated (*r* = 0.74, *p* < 0.0001), indicating that a response strategy that was characterized by a higher number of average presses per round was optimal insofar as it resulted in a higher total payoff. Stated differently, individuals who made few casts on the “sunny-day, catch-and-release” version of the ART failed to optimize their performance and ended up making less on the task than those who pressed more.

Risk-taking propensity, based on total DOSPERT scores, was negatively correlated with ART Total Amount Earned (*r* = −0.53, *p* = 0.01), with a trend for a negative correlation with ART mean Adjusted Presses (*r* = −0.41, *p* = 0.05). In line with our behavioral data—which indicate that an optimal response strategy is characterized by more presses and, as a result, a higher payoff—those individuals that adopted an optimal response strategy (more presses, more earnings) were also lower in self-reported naturalistic risk-taking. Stated differently, individuals who made few casts, and earned less, on the “sunny-day, catch-and-release” version of the ART also report engaging in more risk-taking behaviors. ART performance was not, however, significantly associated with either FI or DI (*p* > 0.05) in our small sample.

Within the BART, the mean Adjusted Presses and Coefficient of Variation were correlated (*r* = −0.42, *p* = 0.05), indicating that participants who pumped more on each balloon were less variable in their response strategy than those who made fewer pumps; this correlation was marginally different between the BART and ART (*p* = 0.06). However, neither of these two BART performance measures was correlated with the Total Amount Earned on the BART. [Note that Adjusted Presses and Total Amount Earned were not perfectly correlated, as our index of Adjusted Presses is the *average* number of balloon pumps across all trials (in rounds that did not result in an explosion); while the total number of Adjusted Presses is identical to the Total Amount Earned on both tasks, the number of presses that the participants makes—on average—across the course of the task provides an index of their strategy and presumably gauges their risk tolerance.] In contrast with the ART, performance on the BART correlated with trait impulsivity: BART Coefficient of Variation negatively correlated with Functional Impulsivity (*r* = −0.52, *p* = 0.01) and Adjusted Presses marginally correlated with Functional Impulsivity (*r* = 0.39, *p* = 0.06), suggesting that individuals high in FI adopted an optimal and consistent response strategy on the task.

Finally, to confirm that our measures of mean Adjusted Presses indexed the general pattern of individual differences in performance across the course of the task (despite variation indexed by Coefficient of Variation), we examined the relationship between Adjusted Presses and the total number of presses across all trials (including Risky-Choice, Cash-out, and Loss trials); note that these Results are not included in Table 4. For both tasks, mean Adjusted Presses was positively correlated with total number of presses across the entire task: ART (*r* = 0.81, *p* < 0.0001) and BART (*r* = 0.51, *p* = 0.01). Furthermore, the pattern of results remained the same when substituting total number of presses for mean Adjusted Presses in relation to Total Amount Earned and Coefficient of Variation (except in one case, where total number of presses was marginally significantly correlated with Total Amount Earned (*p* = 0.056), whereas mean Adjusted Presses was not significantly correlated with Total Amount Earned on the BART). These results suggest that mean Adjusted Presses in each task is a valid indicator of the subject's overall performance across the course of the task, while Coefficient of Variability indexes the degree to which subject's varied from this pattern on Risky-Choice trials across the course of the task.

To summarize, based on an exploratory analysis of the ART behavioral data alone, our data suggest that an optimal performance strategy is reflected in a high number of mean Adjusted Presses as this results in a corresponding high Total Amount Earned. This response pattern is associated with lower rates of self-reported risk-taking, contrary to some previous findings (Pleskac, 2008). Based on the BART behavioral data, participants that pumped more were also less variable in their response strategy, indicating a consistent response strategy. Those individuals that pumped more on the BART, and were less variable in the number of pumps across the task, were also more likely to score high on a self-report measure of functional impulsivity, which is proposed to capture an advantageous response style. Although previous studies have reported an association between increased pumping on the BART and higher self-reported risk taking (Lejuez et al., 2003a,b, 2004, 2007), this is the first investigation of performance and a measure that distinguishes between functional and dysfunctional impulsivity.

#### Individual differences analysis of ART imaging data

**Figure 5:**
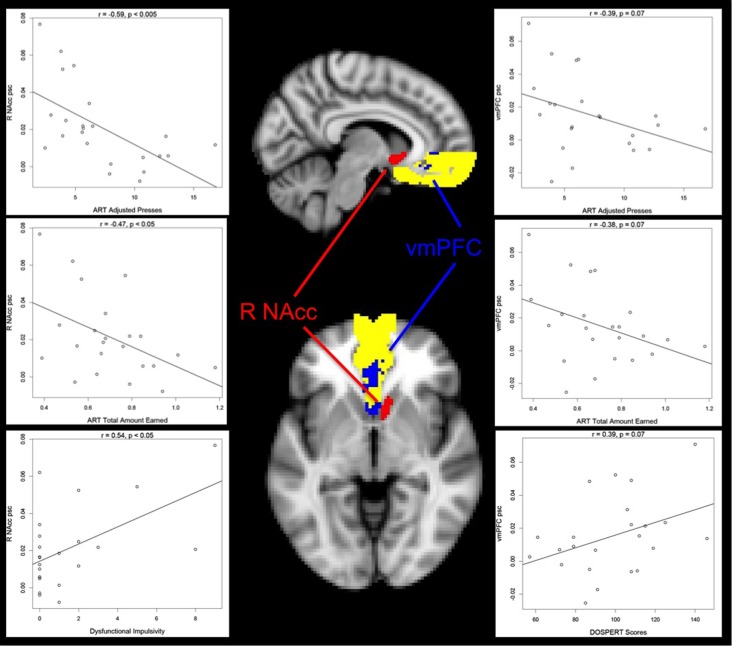
**Relationship between risk-taking performance, impulsivity, risk-taking propensity and Risky choice Parametric activation.** Average percent signal change (psc) values extracted from the right nucleus accumbens (R NAcc) negatively correlate with ART average Adjusted Presses (top left panel) and Total Amount Earned (middle left panel), and positively correlate with Dysfunctional Impulsivity scores (bottom left panel), suggesting that increased reward-tracking activation is associated with poorer performance on the ART and higher impulsivity. A similar pattern is seen in average percent signal change (psc) values extracted from the ventromedial prefrontal cortex (vmPFC), although at trend level: vmPFC activation negatively correlates with ART average Adjusted Presses (top right panel) and Total Amount Earned (middle right panel). vmPFC activation also marginally positively correlates with DOSPERT scores (bottom right panel), suggesting that increased reward-tracking activation is associated with a greater likelihood of engaging in risky behaviors. Activation represents the ART Risky choice Parametric contrast as intersected with our two anatomical ROIs, as described in the text. The vmPFC anatomical mask is presented in yellow, and significant ART Risky choice Parametric activation within this mask is presented in blue. ART Risky choice Parametric activation overlapped with the entire R NAcc mask: these voxels are presented in red. R = Right. For the sagittal view, *X* = 41; for the axial view, *Z* = 34.

To test the hypothesis that reward-related neural activity in a risky setting would be related to individual difference measures, we: (1) extracted percent signal change from anatomically-defined NAcc and vmPFC ROIs for the group Risky choice Parametric contrast; and (2) correlated activation in these ROIs with our individual differences measures. Our individual differences measures included our three primary performance measures (Adjusted Presses, Total Amount Earned, and Coefficient of Variation), trait impulsivity (DI and FI), and naturalistic risk-taking (DOSPERT scores). Percent signal change extracted from the right NAcc was negatively correlated with ART Adjusted Presses (*r* = −0.59, *p* = 0.003, Figure 5, top left panel) and Total Amount Earned (*r* = −0.47, *p* = 0.02, Figure 5, middle left panel), while percent signal change extracted from the vmPFC was marginally negatively correlated with ART Adjusted Presses (*r* = −0.38, *p* = 0.07, Figure 5, top right panel) and Total Amount Earned (*r* = −0.39, *p* = 0.07, Figure 5, middle right panel). These results indicate that greater reward-related neural activity is associated with a suboptimal response strategy in our sample. In line with these findings, percent signal change extracted from the right NAcc was positively correlated with DI (*r* = 0.54, *p* = 0.008, bottom left panel Figure 5), and percent signal change extracted from the vmPFC was marginally positively correlated with total risk-taking (*r* = 0.39, *p* = 0.07, bottom right panel Figure 5), further indicating that greater reward-sensitive neural activity is associated with a self-reported disadvantageous response style and high likelihood of taking risks. For illustration, these relationships are presented in Figure 5 along with a map illustrating the extent of activation in the Risky choice Parametric contrast when intersected with our NAcc and vmPFC anatomically-defined ROIs. The anatomically-defined vmPFC mask is displayed in yellow; activation in the Risky choice Parametric contrast, within this vmPFC ROI, is displayed in blue. The right NAcc mask and Risky choice Parametric activation completely overlapped: this activation is presented in red. Percent signal change in these two ROIs was not significantly associated with any of our other individual differences measures.

#### Individual differences analysis of BART

As a follow-up comparison, percent signal change was extracted from our anatomically-defined ROIs for the BART Risky choice Parametric contrast: while DOSPERT scores negatively correlated with percent signal change extracted for the Risky choice Parametric contrast from the right NAcc (*r* = −0.49, *p* = 0.02), and from the vmPFC (*r* = −0.50, *p* = 0.01), these findings must be considered in light of the fact that this vmPFC activation was not significant in the group contrast and were strictly exploratory.

## Discussion

We conducted an investigation of the ART in a sample of healthy adults in order to characterize the neural correlates of performance on this risky decision-making task, and compare with activation elicited by the BART. The version of the ART used here [named “sunny-day, catch-and-release” in the original paper by Pleskac (2008)] represents decision-making under risk *without* ambiguity, and these results suggest that its decomposable task structure is associated with distinct patterns of neural activation. Specifically, (1) mean activation during Risky choice trials is only seen in right posterior parietal and occipital regions, which is consistent with a comparison of decision-making under risk vs. ambiguity, in which risk preference was correlated specifically with activation in the posterior parietal cortex (Huettel et al., 2006); (2) activation during the Risky choice Parametric contrast (which indexes increasing activation across pumps within a round) is evident in regions known to play a role in reward processing; and (3) activation seen during Cash-out and Loss trials is also consistent with previous reports of corresponding contrasts of the BART task (Rao et al., 2008; Claus and Hutchison, 2012).

In order to examine the specificity of this risk-taking profile, we compared neural activation associated with the ART to that elicited by the BART. We report increased fronto-cingulate activation during BART as compared to ART Risky choice trials, and suggest that this increased activation may be driven by the presence of ambiguity in the BART. This view is supported by previous fMRI investigations of ambiguity, which have demonstrated the recruitment of insular and lateral prefrontal activity with increasing uncertainty (Huettel et al., 2005, 2006; Krain et al., 2006). However, our results do not map precisely onto those of Hsu et al. (2005), who compared conditions of ambiguity vs. risk, and demonstrated that the orbitofrontal cortex, amygdala, and dorsomedial prefrontal cortex were sensitive to ambiguity. This discrepancy may be driven by the qualitative differences in the contrasts of BART vs. ART Risky choice trials and the contrast of ambiguity vs. risk trials as presented by Hsu et al. (2005), or our relative lack of power to detect additional activation sensitive to ambiguity in the BART.

We also report greater activation for the ART as compared to BART Risky choice Parametric contrast, and suggest that this is indicative of tracking of reward, driven in particular by the lack of ambiguity, in the ART. That is, as probability is known and stable across Risky choice trials in the ART, participants are expected to be able to track accumulated reward value; in contrast, Risky choice trials vary in both positive potential gain, loss, and probability of each balloon (represented by an increase in size) in the BART. The only change across the course of Risky choice trials in the ART is the increasing value of accumulated earnings, and this is reflected by significantly greater activation in a valuation network, both as compared to baseline and BART Risky choice Parametric events. We report widespread activation for parametrically weighted Risky choice trials in the ART, but not the BART, through bilateral accumbens and vmPFC extending through other structures of the basal ganglia and cingulate, as well as bilateral lateral prefrontal cortex. These findings are consistent with the known role of these regions in tracking reward value (Jocham et al., 2011; Liu et al., 2011) and are consistent with previous reports of ventral striatal activation when choosing risky options, in contrast to safe or control options, in a decision-making task (Weber and Huettel, 2008). Overall, these results suggest that the ART allows for successful isolation of reward tracking through the course of a sequential risky decision-making task.

In addition to isolating neural correlates of component processes of risky decision-making, our exploratory analyses of individual differences suggest that while ART performance is not associated with trait impulsivity, ART performance is associated with naturalistic risk-taking. In our small sample of healthy adults, a more beneficial strategy—characterized by more presses and a corresponding higher payoff—was associated with less self-reported risk-taking. That is, given the range of responding seen in our sample, a higher number of Adjusted Presses in our sample was reflective of a better response strategy, and not [as originally reported by Lejuez et al. (2002)] indicative of suboptimal decision-making, because in the “sunny-day, catch-and-release” context of this ART version, making a *higher* number of Adjusted Presses was the optimal response strategy. Based on a single index of risk-taking propensity (DOSPERT scores), the Total Amount Earned on the ART was significantly correlated (and mean Adjusted Presses marginally correlated) with naturalistic risk-taking, meaning that participants who performed *better* on this risky decision-making task also viewed themselves as *less likely* to engage in risky behaviors than participants who failed to adopt an optimal response strategy. Stated differently, performance on the ART was sensitive to individual differences in naturalistic risk-taking, with poor performers more likely to engage in naturalistic risk-taking.

Our findings are somewhat in line with those originally reported in the development of the ART (Pleskac, 2008). Specifically, Pleskac compared different version of the ART, including the sunny day (no ambiguity) and cloudy day (with ambiguity) versions and reported a significant correlation between performance in the sunny day version and self-reported risky use of drugs, but no correlation between performance on the cloudy day version and risky drug use. Thus, on the one hand, our findings extend Pleskac's initial conclusion that an ambiguity requirement hinders the predictive validity of the ART. However, Pleskac reported a positive correlation between mean Adjusted Presses and a weighted measure of poly-drug use, whereas we report a negative association between mean Adjusted Presses and self-reported risk-taking across multiple domains of behavior. These sets of findings may suggest that the lack of ambiguity is central to detecting relationships between individual differences in performance and self-report. However, it has also been reported that a fewer number of pumps on the BART is associated with less risk-taking (as indicated by a measure of alcohol use and related problems) (Ashenhurst et al., 2011); this finding is consistent with our results based on the ART, but in contrast to previous reports of the BART (e.g., Lejuez et al., 2002). In addition to differences in the direction of the relationship between task performance and risk-taking, the various indicators of naturalistic risk-taking vary across studies and caution must be used when comparing across such diverse studies; that is, specific measures of poly-drug (Pleskac, 2008) or alcohol use (Ashenhurst et al., 2011) are not equivalent to the composite measure of work-related or personal decisions that involve risk and uncertainty (used here). Overall, these discrepancies indicate that further research on this topic is warranted.

Neural activation during performance of the ART was associated with both naturalistic risk-taking and trait impulsivity. During ART Risky choice trials, activation from the right NAcc and vmPFC increased with reward values across the course of the task. *Increased* activation in these regions was related to *suboptimal task performance* (lower than optimal number of Adjusted Presses and less money earned), *higher dysfunctional impulsivity*, and a *higher risk-taking propensity*. Our results therefore suggest that activation associated with increasing reward in the ART is associated with poorer decision-making performance in this risky context and with a greater likelihood of engaging in risky behaviors. These regions (NAcc and vmPFC) form part of a valuation network, which is engaged during reward-processing across a range of paradigms (for a review, see Liu et al., 2011), and shows an increase in activation as a function of both reward magnitude (Knutson et al., 2001; Peters and Buchel, 2010) and impulsivity (Hariri et al., 2006; Hahn et al., 2009; Jimura et al., 2013). While preliminary, these findings illustrate a specific context in which behavior, trait impulsivity, and self-reported naturalistic risk-taking are correlated, and may help to clarify mixed findings regarding the relationship between impulsivity and decision-making task performance (broadly defined) (Zermatten et al., 2005; Perales et al., 2009; Billieux et al., 2010; Bayard et al., 2011).

These findings of a relationship between reward-related activation and DI, as well as risk-taking propensity, are consistent with previously reported associations between both risk-taking traits and behaviors with activation in brain regions engaged during risky decision-making: specifically, ventral striatal response during reward anticipation and receipt has been positively associated with sensation-seeking scores in healthy adolescents (Bjork et al., 2008), risk-taking propensity in healthy adolescents (Galvan et al., 2007), and psychopathic personality traits, particularly an impulsive-antisocial factor, in adults (Buckholtz et al., 2010). Similarly, medial prefrontal activation during reward processing has been positively associated with psychosocial symptoms on a Drug Use Screening Inventory in healthy adolescents (Bjork et al., 2011). However, we demonstrate that pattern here with a task that is also decomposable into component processes underlying decision-making. Taken as a whole, these findings suggest that individuals that are more sensitive to reward—as indexed by heightened ventral striatal and medial prefrontal activation when tracking reward, as well as higher DI and self-reported risk-taking—perform suboptimally in the face of unambiguous reward. We speculate that the enhanced reward-related neural activity is part of an imbalance between systems underlying decision-making, as several recent lines of evidence demonstrate altered connectivity between valuation and control networks (Cox et al., 2010; Helfinstein et al., under review), as well as less online, but more offline, activity (Hahn et al., 2011; Shannon et al., 2011) in individuals with high impulsivity or risk-taking propensity. It is therefore possible that ventral striatal and medial prefrontal activity is biased toward tracking of reward in certain individuals, and that this bias underlies suboptimal decision-making (as measured using the ART), predisposing an individual to act with less forethought (DI) and an increased likelihood of engaging in high-risk behaviors (as measured by the DOSPERT). This is speculative insofar as we are inferring that high ventral striatal and medial prefrontal activation elicited by the Risky-choice Parametric contrast is specific to reward processing in the context of a complex decision-making task.

One major limitation of our primary analyses is that all participants completed the ART after having completed the BART, although the time between scans varied across participants [mean time difference in days was 229.87 (*SD* = 181.75)] and the time difference between scans was not correlated with any variable examined in our analyses. The wide range of task parameters used in the literature, for both BART and ART, makes a comparison to previously reported results difficult. However, the distribution of performance summary measures reported here is relatively consistent with those reported previously (e.g., Lejuez et al., 2002; Pleskac, 2008), suggesting that performance strategies did not drastically shift between testing sessions. Nonetheless, the influence of order effects cannot be excluded. Furthermore, the tasks differ in important ways: the tasks involve different metaphors, participants played for points in the BART, while they played for pennies in the ART, and the BART task alone included a control condition. While risk is explicit and stable in the ART, the potential increasing reward, loss, and probability of risk varies across BART trials, making it difficult to separate out which of these parameters is driving neural correlates of what we are labeling as ambiguity here. Thus, while the current investigation has attempted to compare tasks that differ on a set of hypothesized component processes (e.g., ambiguity), we cannot rule out additional differences in task parameters that may account for our results.

There are significant limitations to acknowledge in our exploratory analyses: in particular, as the small sample size leaves us underpowered, the reported associations are not corrected for multiple comparisons. Our goal in these exploratory analyses was to supplement our primary analyses of the ART, and comparison of the ART and BART. This is the first examination of the neural correlates of the ART and as such, we sought to help with the interpretation of increasing Risky Choice parametric activation by examining its relationship with between task performance, individual difference measures of impulsivity, and self-reported risk-taking. Although underpowered and exploratory, and although the absence of significant relationships may also be driven by a lack of power, we offer these results as preliminary evidence in support of a distinct risk-taking profile that may be better captured by the ART, and hope to stimulate additional research. Another limitation is the use a single measure of risk-taking propensity (i.e., DOSPERT total risk-taking propensity vs. subscale scores), given the lack of evidence suggesting differences in ART or BART performance as a function of risk-taking across domains (e.g., Recreational vs. Social) and given the relatively small sample size. This represents an area for follow-up investigation and evidence characterizing differences as a function of risk-taking across domains will further contribute to the characterization of how individual differences in impulsivity and risky decision-making relates to naturalistic risk-taking. Overall, additional work in larger samples characterized by a broader range of trait impulsivity is needed to characterize how the task parameters of both the ART and BART relate to individual differences in trait impulsivity and naturalistic risk-taking.

Results reported here suggest that the ART, which is decomposable and associated with distinct patterns of neural activation, is also associated with individual differences in trait DI and naturalistic risk-taking. The lack of ambiguity, paired with the presence of affective features, enables participants to track reward value across trials explicitly, which is most evident in those who have a heightened sensitivity to reward. This reward sensitivity correlates with self-reported DI and a global measure of self-reported naturalistic risk-taking propensity. In contrast, our results suggest that the presence of ambiguity in the BART obscures a participant's ability to assign potential accumulated reward, and as a result, elicits a suboptimal response strategy—all of which mask the relationship between performance and naturalistic risk-taking. As performance on the ART—but not BART—is associated with self-reported naturalistic risk-taking, this characterization of the ART represents a step toward identifying a risk-taking profile, and of being able to use behavioral measures of risky decision-making to predict naturalistic risk-taking. These findings are in line with the overarching goal of this line of research, which is to identify mechanisms that predispose an individual to high-risk behaviors, thereby enabling the prediction, and in turn, the prevention, of risk behaviors that carry high levels of morbidity and mortality (e.g., fast driving) or that contribute to the symptomatology of clinical illness (e.g., substance dependence). However, our small sample requires that caution must be used when drawing conclusions based on these initial results and future studies are needed to replicate and extend these findings.

## Author contributions

Designed the study: Eliza Congdon and Russell A. Poldrack. Collected data: Eliza Congdon, Angelica A. Bato, Katherine H. Karlsgodt, Fred W. Sabb. Analyzed the data: Eliza Congdon, Angelica A. Bato, Jeanette A. Mumford. Contributed to task implementation: Tom Schonberg. Contributed data and manuscript input: Edythe D. London, Tyrone D. Cannon, Robert M. Bilder. Wrote the paper: Eliza Congdon, Angelica A. Bato, Tom Schonberg, Jeanette A. Mumford, Russell A. Poldrack.

### Conflict of interest statement

The authors declare that the research was conducted in the absence of any commercial or financial relationships that could be construed as a potential conflict of interest.
